# Enhanced Detection of *Vibrio harveyi* Using a Dual-Composite DNAzyme-Based Biosensor

**DOI:** 10.3390/bios14110548

**Published:** 2024-11-13

**Authors:** Siying Li, Shuai Zhang, Weihong Jiang, Yuying Wang, Mingwang Liu, Mingsheng Lyu, Shujun Wang

**Affiliations:** 1 Jiangsu Key Laboratory of Marine Bioresources and Environment/Jiangsu Key Laboratory of Marine Biotechnology, Jiangsu Ocean University, Lianyungang 222005, China; syingli@jou.edu.cn (S.L.); 2022210128@jou.edu.cn (S.Z.); weihongjiang@jou.edu.cn (W.J.); yuyingw@jou.edu.cn (Y.W.); mwliu@jou.edu.cn (M.L.); mslyu@jou.edu.cn (M.L.); 2 Co-Innovation Center of Jiangsu Marine Bio-Industry Technology, Jiangsu Ocean University, Lianyungang 222005, China

**Keywords:** *Vibrio harveyi*, dual DNAzyme, SELEX, field detection

## Abstract

*Vibrio harveyi* is a serious bacterial pathogen which can infect a wide range of marine organisms, such as marine fish, invertebrates, and shrimp, in aquaculture, causing severe losses. In addition, *V. harveyi* can be transmitted through food and water, infecting humans and posing a serious threat to public safety. Therefore, rapid and accurate detection of this pathogen is key for the prevention and control of related diseases. In this study, nine rounds of in vitro screening were conducted with Systematic Evolution of Ligands by Exponential Enrichment (SELEX) technology using unmodified DNA libraries, targeting the crude extracellular matrix (CEM) of *V. harveyi*. Two DNAzymes, named DVh_1_ and DVh_3_, with high activity and specificity were obtained. Furthermore, a fluorescent biosensor with dual DNAzymes was constructed which exhibited improved detection efficiency. The sensor showed a good fluorescence response to multiple aquatic products (i.e., fish, shrimp, and shellfish) infected with *V. harveyi*, with a detection limit below 11 CFU/mL. The fluorescence signal was observed within 30 min of reaction after target addition. This simple, inexpensive, highly effective, and easy to operate DNAzymes biosensor can be used for field detection of *V. harveyi*.

## 1. Introduction

*Vibrio harveyi* is a curved rod-shaped, halophilic, Gram-negative bacterium which is widely distributed in marine and estuarine aquatic ecosystems [1]. It is a major causative agent of infections in marine fishes, shellfish, and crustaceans, especially in the warm waters of Asia, Southern Europe, and South America [2]. With increasing numbers of aquaculture species in the world, the range of hosts infected by this pathogen is also expanding gradually. Fish infected with this pathogen generally show symptoms such as scale shedding, muscle necrosis, vasculitis, infectious necrotizing enteritis, eye lesions, etc. [3]. The infection can also lead to gill rot, red-body disease, *Vibrio luminescens* disease (in shrimp) and wrinkled disc abalone pustulosis [4], muscle atrophy, and square-speckled eastern snail swollen kiss, etc. (in shellfish) [5]. Due to its wide prevalence and high mortality rate, *V. harveyi* infection has caused great economic losses to aquaculture industry [6]. Meanwhile, *V. harveyi* has emerged as a foodborne pathogen, causing acute gastroenteritis and inflammation of infected wounds, and even leading to subcutaneous tissue and muscle necrosis in humans [7]. Thus, it poses serious threats to the quality and safety of aquatic products and human health. Currently, there is no effective treatment for *V. harveyi*, other than antibiotics [7]. Therefore, early detection of *V. harveyi* is particularly important for prevention and control of related diseases. The traditional microbial culture is a cumbersome and time-consuming method, which is inefficient for timely detection of bacterial infections [8]. PCR assay has the advantages of high specificity and sensitivity, but it is expensive, requires precise temperature control and temperature circulators, and may lead to false-positive results. Immunological tests are less prone to false-positive results, but require specialized technicians and expensive instruments and reagents [9]. These factors limit the applicability of these methods in field testing. Therefore, there is a need to develop a simple and efficient method for rapid detection of bacterial infection.

Deoxyribozymes (DNAzymes) are DNA oligonucleotides that exhibit superior catalytic activity in the presence of cofactors [10]. A DNAzyme with specific catalytic activity for a target substrate can be isolated from billions of candidate DNA sequences by an exponentially enriched ligand systematic evolution technique (SELEX) [11]. A DNAzyme usually consists of a substrate strand and an enzyme strand, which acquire catalytic function by complementary base pairing, forming a double-stranded system and folding into certain two- and three-dimensional spatial structures [12]. The presence of a target activates the DNAzyme, resulting in cleavage of nucleotides (rA) on the substrate strand. A variety of DNAzymes with different catalytic properties have been screened, such as RNA cleavage DNAzyme, DNA cleavage DNAzyme, and DNAzyme catalyzing ligation reactions. In a previous study, a DNAzyme biosensor was cleverly designed to conduct and amplify signals through conformational changes and cascade reactions, facilitating highly sensitive detection [13]. In addition, DNAzyme is commonly used as a design element for biosensors due to its unique properties, such as excellent chemical stability, ease of synthesis and modification, high binding affinity, and specificity. DNAzyme can not only be conveniently used for signal amplification (such as nucleic acid amplification), but also can be easily integrated into analytical reporting systems, such as fluorescence and colorimetric and electrochemical methods using various signal transduction mechanisms [14]. Due to these advantages, DNAzyme has broad application prospects in food regulation, environmental monitoring, and clinical medical research. It is widely used for the detection of metal ions, nucleic acids, and bacteria, and serves as an ideal molecular tool for the development of sensitive and efficient on-site detection methods.

The DNAzyme was first reported in 1994, when Breaker and Joyce obtained a DNA sequence through in vitro screening of a large number of random DNA sequences using the SELEX technique. The obtained DNA sequence could cleave RNA at a specific site, and it catalyzed a Pb^2+^ dependent cleavage reaction of RNA phosphates [15]. Afterwards, DNAzymes working with various metal ions such as Zn^2+^ [16], Hg^2+^ [17], Ag^+^ [18], Cu^2+^ [19], Mg^2+^ [20] and UO_2_^2+^ [21] were screened one after another, exhibiting good catalytic properties. With this deepening of research, DNAzymes were applied in microbiological testing, clinical practice and medical research. In 2013, in China, Li et al. utilized DNAzyme in bacterial detection (*Escherichia coli*) for the first time [22]. Ali et al. constructed a portable paper-based sensor for detection of *Helicobacter pylori* using DNAzyme and provided a new idea for rapid detection of foodborne pathogens [23]. In addition, a number of RNA cleavage fluorescents DNAzyme (RFDs) have been isolated for detection of various pathogens, including *Clostridium difficile* [24], *Legionella pneumophila* [25], *Vibrio Anguillarum* [26], *Klebsiella pneumoniae* [27], *Vibrio cholera* [28], and *salmonella* [29]. In addition to the detection of heavy metals and bacterial pathogens, DNAzyme has also been used for detection of toxic gases, such as hydrogen sulfide (H_2_S) [30], in the environment, as well as for rapid detection of new coronavirus SARS-CoV-2 protein [31], ochratoxin A [32], Nipah virus [33], and Zearalenone [34]. Furthermore, combinations of DNAzyme and nanomaterials have been used in many biosensors and clinical medicine applications, such as targeted drug delivery [35] and cancer treatment [36].

In 2020, Setlem reported a dual aptamer-DNAzyme based colorimetric assay for detection of AFB_1_ in complex samples [37]. In this experiment, DNAzyme-ligated aptamers were used to detect AFB_1_ captured by apta magnetic beads, and the colorimetric signals were detected and quantified with high stability and specificity. Based on this experiment, a dual DNAzyme biosensor has been designed in this study for detection of *V. harveyi*. Firstly, a DNAzyme that could specifically recognize *V. harveyi* was screened using SELEX technology, and then the reaction conditions were optimized to prepare a DNAzyme biosensor with high specificity and good sensitivity. This study will provide a simple and rapid method for detection and identification of *V. harveyi* in aquatic products. The findings of this study would provide a technical basis for the detection of other aquatic pathogens and could promote the healthy and stable development of the aquaculture industry.

## 2. Materials and Methods

### 2.1. Chemicals and Bacterial Strains

*V. harveyi*, *Pseudomonas fluorescens*, *Vibrio cholerae*, and *Edwardsiella tarda* strains were purchased from the China Industrial Microbial Strain Conservation and Management Center (CICC). *Aeromonas salmonicida* and *Aeromonas hydrophila* were supplied by Jiangsu Key Laboratory of Marine Bioresources and Environment. DNA libraries, primers, and 3′,6′-dihydroxy-3-oxopyranosyl-isobenzofuran (FAM)-labeled substrates used for in vitro screening, as well as streptavidin-coated magnetic particles and sequences of sensors were purchased from Sangon Bioengineering Co., Ltd. (Shanghai, China). Reagents, including Tween-20, sodium hydroxide, potassium dihydrogen phosphate, potassium chloride, and anhydrous ethanol, were purchased from Sinopharm Chemical Reagent Co., Ltd., Shanghai, China. While gel-red nucleic acid dye (10,000×) and Marker (25~500 bp) were purchased from Sangon Bioengineering Co., Ltd., Shanghai, China. Gel-carrying blue dye (6×) was purchased from Biolabs (Cambridge, MA, USA). Taq DNA polymerase, 40% acrylamide solution (29:1), RNase A, RNase H, and RNase T1 were purchased from Sangon Bioengineering Co., Ltd., Shanghai, China. All solutions were prepared using ultrapure water (resistance: >18.20 MΩ × cm) treated by a Bamstead Labtower EDI water purifier (Thermo Fisher, Rheinland-Pfalz, Germany) and filtered by a 0.22 μM filter membrane.

### 2.2. Bacterial Culture and Preparation of Extracellular Products

*V. harveyi* was inoculated into 25 mL of liquid Luria-Bertani (LB) medium consisting of 1% tryptone, 0.5% yeast powder, and 1% NaCl, and with pH 7.0. The inoculated media were incubated at 28 °C and 180 rpm for 12–15 h. At the same time, a three-zone delimitation culture of suspension was carried out on solid LB medium to detect any contamination in the bacterial suspension. After confirming the absence of contamination, 500 μL of *V. harveyi* preserved in glycerol was inoculated into 25 mL of liquid LB medium. The absorbance of bacterial suspension was recorded at a wavelength of 600 nm, and the incubation was stopped when it was close to 1. A portion of bacterial culture solution was transferred to centrifuge tubes (1.5 mL) and centrifuged at 13,000× *g* for 5 min at room temperature. The supernatant was collected and stored in a refrigerator at −20 °C. Another portion of bacterial culture solution was diluted (10^−9^) using saline gradient. The diluted culture was poured onto solid LB medium (100 μL), and the media plates were incubated at 28 °C for 48 h. After incubation, bacterial colonies were counted to determine the number of bacteria per milliliter. Crude extracellular matrix (CEM) was obtained from five bacteria (i.e., *P. Fluorescens*, *V. cholerae*, *E. tarda*, *A. salmonicida*, and *A. hydrophila*) after culturing at their respective optimal growth temperatures using the method described above. The collected CEM samples were stored in a refrigerator at −20 °C.

### 2.3. Preparation of DNA Libraries

In this study, SELEX technology was utilized for in vitro screening. Firstly, forward and reverse primers, as well as random DNA libraries, were designed and synthesized by Bioengineering Co., Ltd., Shanghai, China. The details of the oligonucleotide sequences have been provided in Table 1. Oligonucleotide sequences of forward primers, which contained a biotin tag and adenylic acid (named as rA), were ligated into the libraries by PCR. The forward primer and rA were used as substrates for the DNA libraries and cleavage junctions, respectively.

The Lib-N40-pool and primers were amplified by PCR to prepare an initial DNA library which met the cleavage requirements. During the first round of selection, the synthesized DNA library was ligated to adenine ribonucleotide (rA) through PCR. The PCR reaction mix consisted of a 20–50 ng/µL DNA library (1 µL), 10 µmol/L forward and reverse primers (2 µL each), 25 mmol/L MgCl_2_ (2 µL), 1.25 U/µL Taq DNA polymerase (25 µL), and sufficient double-distilled H_2_O (ddH_2_O) to constitute a total volume of 50 µL. PCR amplification conditions were as follows: 95 °C, 3 min; 95 °C, 15 s; 58.5 °C, 15 s; 72 °C, 1 min, and, finally, 72 °C for 5 min. The optimal number of thermal cycles was determined by conducting the amplification reaction for 7, 9, 11, 13, 15, 17, 19, 21, 23, and 25 cycles. PCR library enrichment was carried out with the optimal number of cycles. The obtained PCR products were purified by 3% agarose gel electrophoresis for further experiments.

### 2.4. Screening of DNAzyme

**Figure 1:**
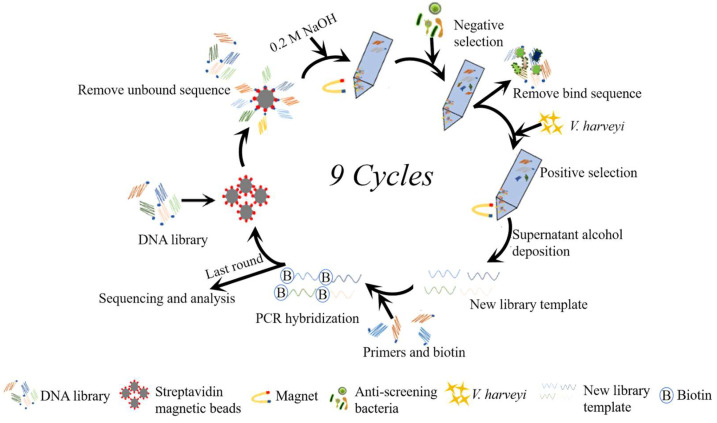
Flowchart of DNAzyme screening. The library contained 40 random nucleotides and was screened for nine rounds. Negative selection was carried out during the 5th and 7th rounds of screening, while positive selections were conducted in the other rounds. Biotin was labeled at the 5′ end. The target molecule was the CEM of bacteria.

The screening process is shown in Figure 1. The DNA library obtained after PCR contained biotin, which could bind to streptavidin-coated magnetic beads. Negative selection of DNA libraries was conducted by incubation with other bacterial CEMs (*P. Fluorescens*, *V. cholerae*, *E. tarda*, *A. salmonicida*, and *A. hydrophila*). The cleaved DNA was removed, and the undeleted DNA library was collected for the next round of forward screening. The DNA (with a specific structure), combined with CEM of *V. harveyi* (CEM-Vh), could then undergo cleavage reaction, and the result was used in the next round of screening. The active sequence was efficiently amplified by PCR and enriched in the next round of selection. A total of nine rounds of screening were performed. Among them, rounds 1, 2, 3, 4, 6, 8, and 9 were for positive selection, while the remaining two rounds aimed to accomplish negative selection. The products obtained after the ninth round of alcohol precipitation and drying were enriched by the PCR library. The enriched product was recovered and sent to Sangon Bioengineering Co., Ltd. (Shanghai, China) for sequencing.

### 2.5. Screening of Candidate DNAzyme Activity

The top six sequences showing the highest enrichment rate after high-throughput sequencing were used as candidate DNAzymes. The candidate DNAzymes and substrate were synthesized by Sangon Bioengineering Co., Ltd., Shanghai, China. After combining the candidate DNAzyme sequences with substrate, their cleavage activities were compared by denatured polyacrylamide gel electrophoresis (dPAGE) and fluorescence detection. DNAzyme complex (namely DVh-S) was prepared by heating the mixture of 5 μM substrate, 7.5 μM DNAzyme, and 2× selection buffer (100 mM HEPES, 300 mM NaCl, 30 mM MgCl_2_, and 0.02% Tween 20, pH 7.5) in a boiling water bath for 3 min under dark conditions and then cooling it to room temperature. Throughout this process, the fluorescent FAM labeled at the 5′ end of the substrate chain was completely quenched by the quenching group at the 3′ end of DNAzyme to prevent the appearance of false fluorescence. A total of 4 μL of DVh-S, 45 μL of 2× selection buffer, and 10 μL of CEM-Vh were added to light-proof the 96-well plate. The solutions were shaken and mixed, and then sufficient ddH_2_O was added to make up 100 μL of each solution. Fluorescence intensity signals of six candidate DNAzyme were monitored for 2 h at regular intervals of 30 s (excitation wavelength: 485 nm, emission wavelength: 535 nm) using a fluorescent enzyme spectrometer (Infinite M1000Pro, Tecan, Männedorf, Switzerland).

To further verify the cleavage activity of the DNAzyme, 4 μL of DVh-S, 25 μL of 2× selection buffer, and 10 μL of CEM-Vh were supplemented with ddH_2_O to constitute a volume of 50 μL. After conducting the reaction under dark conditions for 20 min, 2× gel loading dye blue (containing 8 M urea) was added to terminate the reaction. After the reaction, each well was rinsed with 1× TBE to flush out the precipitated urea. Volumes of 15 μL of the sample mixture and blank control were resolved by conducting gel electrophoresis at 150 V for 30 min. The resolved gel was visualized and quantitatively analyzed (cleaved DNA + uncleaved DNA = 100%) using the Bio-Rad GelDoc™ EZ imaging system (BIO-RAD, Hercules, CA, USA).

Based on the above results, DVh_3_, with the highest activity, was selected and combined with DVh_1_ and DVh_5_ to construct a dual DNAzyme system. This system was used to jointly capture the targets with improved detection efficiency. The specific operation was as follows: 4 μL of DVh_3_-S was combined with 2 μL of DVh_1_-S and 2 μL of DVh_5_-S in a light-proof 96-well plate. Subsequently, 45 μL of 2× selection buffer and 10 μL of CEM-Vh were added and mixed, and then sufficient ddH_2_O was added to make up a volume of 100 μL. The reaction was carried out for 1 h, and the fluorescence signals were recorded.

### 2.6. Optimization of Reaction Conditions

#### 2.6.1. pH

Selection buffers with different pH (4.5–10.0) were used to determine the optimal pH. The selection buffer consisted of 100 mM of HEPES (4-(2-hydroxydiformyl) piperazine-1-dimethylsulfonic acid), 300 mM of NaCl, 30 mM of MgCl_2_, and 0.02% of Tween-20. The pH of the 2× S buffer was adjusted using HCl and NaOH. Thus, the reactions between DVh_3+1_ and CEM-Vh were conducted at different pH, and the intensities of fluorescence signals were compared.

#### 2.6.2. Metal Ions

The most common monovalent ion Na^+^ and eight bivalent metal ions (Sr^2+^, Co^2+^, Fe^2+^, Zn^2+^, Mg^2+^, Ba^2+^, Mn^2+^, and Ca^2+^, with the final concentration of 30 mM and optimal pH) were selected for the preparation of the buffer solution, with the addition of EDTA. This mixture was used as the control group in this experiment. Quantities of 4 μL DVh_3_-S, 2 μL DVh_1_-S, 45 μL different ion buffers, 41 μL ddH_2_O, and 10 μL CEM-Vh were mixed properly. The fluorescence intensity signal of the solution was monitored for 1 h through fluorescent enzyme labeling. Furthermore, concentrations of the best bivalent metal ion (i.e., Mg^2+^) and the only univalent ion, Na^+^, were optimized by comparing the influence of different concentrations of Mg^2+^ and Na^+^ (0, 30, 60, 90, 120, 150, 180, 210, 240, 300, and 400 mM) on the cleavage activity of DVh_3+1_ relative to the fluorescence intensity signal.

### 2.7. Specificity of Dual DNAzyme

To determine the specificity of the DNAzyme, the dual DNAzyme (DVh_3+1_) with the best cleavage activity was allowed to react with CEM-Vh and other bacterial CEMs, as described in Section 2.5. Fluorescence intensity monitoring and dPAGE experiments were performed to verify the cleavage activity of the dual DNAzyme.

### 2.8. Sensitivity Detection

A quantity of 100 μL of initial culture medium was taken into centrifuge tube and mixed with 900 μL of normal saline for a 10-fold gradient dilution (10^0^–10^9^). Then, 4 μL DVh_3_-S (5 μM substrate + 7.5 μM DVh_3_), 2 μL DVh_1_-S (5 μM substrate + 7.5 μM DVh_1_), and 39 μL ddH_2_O were added to the 96-well plate. Subsequently, 45 μL 2× selection buffer and 10 μL diluted bacterial solution were added. The total reaction volume was 100 μL. The reactions were performed for 2 h and fluorescence signals were monitored. Subsequently, the sample of reaction solution was subjected to dPAGE analysis.

### 2.9. Identification of the Target and Its Molecular Weight

According to previous reports, the targets recognized by DNAzyme are mostly proteins [23]. Therefore, CEM-Vh was cultured at 37 °C for 30 min in presence of five types of proteases (i.e., proteinase K, pepsin, trypsin, bromelain, and alkaline protease at a ratio of 1:1) to completely dissolve the proteins in CEM-Vh; the fluorescence intensity signals of these cultures were monitored for 2 h.

The proteins secreted by *V. harveyi* have been reported to have a molecular weight of between 25.0 to 66.2 kDa [38]. Therefore, three filter membranes, with pore sizes 10, 30, and 50 kDa, were selected for the ultrafiltration of CEM-Vh. CEM-Vh samples with four molecular weights were used as targets during fluorescence monitoring and dPAGE experiments.

### 2.10. Influence of RNases on Cleavage Activity of DNAzyme

The influence of RNases was assessed to ensure that the cleavage of target was that specifically performed by the DNAzyme, rather than a non-specific cleavage caused by RNase. Four common RNases (RNase A, RNase I, RNase H, and RNase T1) were acquired from Thermo Scientific^TM^ (Waltham, MA, USA). Quantities of 2 μL of each RNase were each added to 198 μL ddH_2_O for dilution. Quantities of 4 μL DVh_3_-S, 2 μL DVh_1_-S, 29 μL ddH_2_O, and 35 μL of 2× selection buffer were added to the light-resistant 96-well plate. The wells were divided into five groups: control, RNase A, RNase H, RNase I, and RNase T1. A quantity of 30 μL ddH_2_O was used as blank control, while RNase A, RNase H, RNase I, RNase T1, and CEM-Vh were added into their respective experimental groups. The fluorescence intensity signals were measured for 1 h.

### 2.11. Design and Optimization of Dual DNAzyme Sensors

Using the transparent lid of the 96-well plate as the mold, DNAzyme sensors were secured in the shallow round holes in the plate. Dvh_3_-S and DVh_1_-S were mixed (2:1) and labelled as DVh_3+1_-S. Quantities of 5 μL DVh_3+1_-S, 25 μL 2× selection buffer, 10 μL pullulan polysaccharide (8%), and 10 μL of 0.25 M trehalose (the prepared trehalose was stored away from light) were poured into a dark tube and mixed. In all, 30 μL of this solution was added into the shallow circular holes of the 96-well plate. The plate was covered with tin foil and heated in an oven at 50 °C under dark conditions for 20 min. The wells were divided into experimental and control groups. A total of 30 μL of CEM-Vh was added in the experimental group, while 30 μL ultrapure water and 30 μL culture medium were added into the wells of the two blank groups, respectively. The fluorescence intensity signals were monitored by fluorescent enzyme labeling.

Furthermore, the concentration of DVh_3+1_-S in the sensor was optimized by comparing the fluorescence intensity signals of DVh_3+1_-S at different concentrations (50, 100, 200, 300, and 400 nM).

### 2.12. Detection of V. harveyi in Actual Samples by the Dual DNAzyme Sensor

Healthy *Cyrenodonax formosana*, *Epinephelus, Penaeus vannamei*, and whelk were placed in a 20-L seawater tank at 16 °C with continuous aeration. Two hundred microliters of *V. harveyi* bacterial culture (OD_600_ was about 0.8), incubated at 28 °C for 12 h, was injected intramuscularly into the aquatic organisms of the experimental group, while 200 μL sterile normal saline was injected into the organisms of the control group. Three parallel doses were given to the organisms in each group. During the experiment, these organisms were observed regularly. Livers of animals that died were collected, ground, and centrifuged with normal saline. The liver cells were then cultured at 28 °C in thiosulfate citrate bile salts sucrose agar culture medium (TCBS).

The bacteria isolated from the liver samples of the four organisms were sent to Sangon Bioengineering Co., Ltd., Shanghai, China, for 16S rDNA sequencing. The bacterial sequences were compared with the sequence of *V. harveyi*. Liver samples of *Cyrenodonax formosana*, *Epinephelus, Penaeus vannamei*, and whelk were used as bacterial sources. A quantity of 30 μL of the supernatant of each liver sample was added to the optimized sensor and the fluorescence intensity signal was recorded for 30 min. The sample with the highest fluorescence intensity signal was diluted ten times, and then 30 μL diluent was added to the sensor. Fluorescence intensity was recorded for 30 min, and linear fitting analysis was performed to determine the detection limit of sensor.

### 2.13. Data Analysis

All of the experiments were conducted on three parallel samples, and the data were analyzed by SPSS v20. The bars or dots in the figures represent mean ± SD. Significant differences (*p* < 0.05) were marked as different letters.

## 3. Results

### 3.1. Screening of DNAzyme Activity

**Figure 2:**
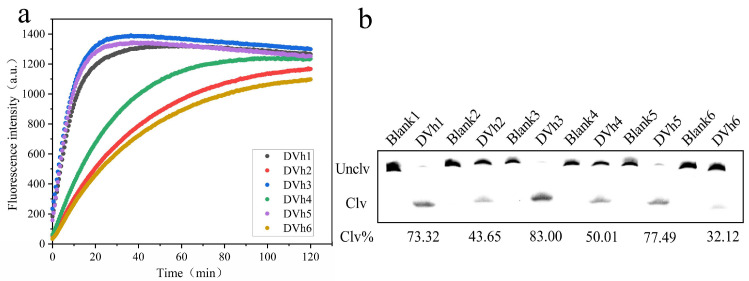
Activity of candidate DNAzymes: (**a**) fluorescence intensity (DVh1–6 reacted with CEM-Vh); and (**b**) results of candidate 15% dPAGE (DVh1–6 reacted with CEM-Vh, Blank1–6 mean DVh1–6 reacted without CEM-Vh).

After nine rounds of screening, a total of 91,038 original DNA sequences were obtained by high-throughput sequencing, and 6 DNA sequences were selected, according to the sequence length and percentage from high to low, and named as DVh_1_, DVh_2_, DVh_3_, DVh_4_, DVh_5_, and DVh_6_, respectively (Table 2). Fluorescence analysis and gel electrophoresis experiments were used to compare the activities of these six candidate DNAzymes. As shown in Figure 2, all six DNAzymes possessed cleavage activity for CEM-Vh, with DVh_1_, DVh_3_, and DVh_5_ showing higher fluorescence signals and larger percentages of cleavage bands (i.e., 73.32%, 83.00%, and 77.49%, respectively). Therefore, DVh_3_, with the highest activity, was selected as the base DNAzyme, while DVh_1_ and DVh_5_ were used as auxiliary DNAzymes to construct a dual DNAzyme system that could jointly capture targets to improve detection efficiency.

**Figure 3:**
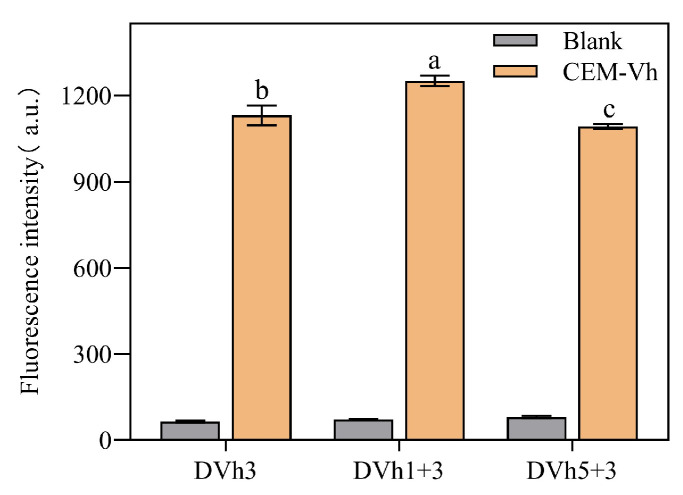
Differences between the fluorescence intensity signals of single and dual DNAzymes. (Blank means without adding CEM-Vh, CEM-Vh means adding CEM-Vh. Different letters indicate statistically significant differences (*p* < 0.05), while identical letters indicate insignificant differences (*p* > 0.05)).

At the same concentration, the fluorescence intensity signal of dual DNAzyme (DVh_3+1_) was significantly higher than those of the other two DNAzymes (Figure 3). Therefore, DVh_3+1_ was selected for the subsequent experiments.

### 3.2. Optimization of DVh_3+1_ Reaction Conditions

**Figure 4:**
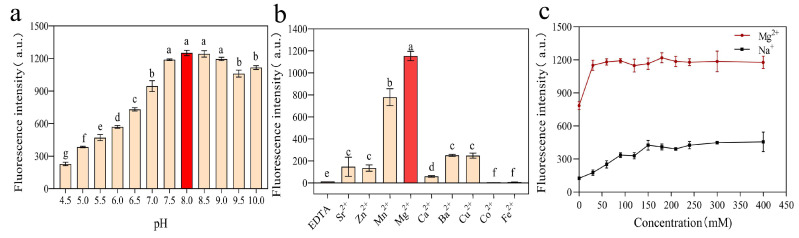
Optimization of reaction conditions: (**a**) pH optimization; (**b**) influence of various divalent metal ions on the cleavage activity of DVh_3+1_ (different letters indicate statistically significant differences (*p* < 0.05), while identical letters indicate insignificant differences (*p* > 0.05)); and (**c**) optimization of the concentrations of Na^+^ and Mg^2+^. Buffer/EDTA reaction contained 300 mM EDTA in 2× selection buffer. The bars and the dots represent mean ± SD.

As shown in Figure 4a, DVh_3+1_ showed the highest cleavage activity at pH 8.0. Therefore, subsequent experiments were performed using buffer solution with a pH of 8.0. EDTA can form a stable soluble metal complex with divalent ions. As shown in Figure 4b, DNAzyme could not exhibit any significant cleavage activity without the assistance of divalent metal ions (EDTA group). This indicated the influence of divalent metal ions on the cleavage activity of the DNAzyme. At the same concentration, the fluorescence intensity signal was the highest in the presence of Mg^2+^. Therefore, Mg^2+^ was considered the best divalent ion and used in subsequent experiments. Furthermore, concentrations of Mg^2+^ and the most common monovalent ion, Na^+^, were optimized. As shown in Figure 4c, the cleavage activity of DVh_3+1_ was the highest at a Na^+^ concentration of 300 mM. Therefore, 300 mM was determined to be the optimal concentration of Na^+^. Similarly, the optimal concentration of Mg^2+^ was observed to be 180 mM.

### 3.3. Specificity Analysis of DVh_3+1_

**Figure 5:**
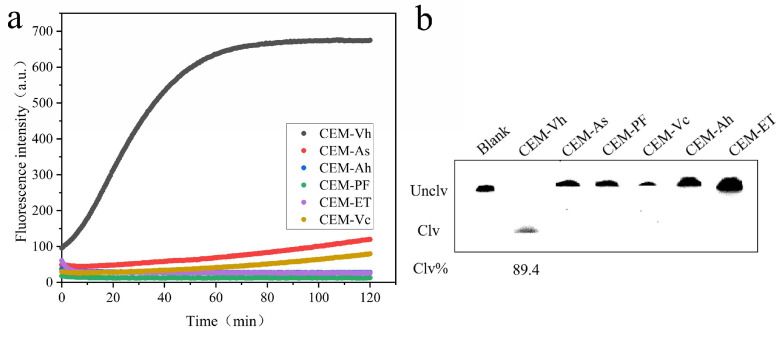
Specificity of DVh_3+1_: (**a**) fluorescence intensity of DVh_3+1_ in presence of CEM of various bacteria; (**b**) specificity of DVh_3+1_ analyzed by 15% dPAGE (Blank: reaction system without CEM-Vh).

Five common aquatic pathogens were used as control samples, and the specificity of DVh_3+1_ was evaluated by fluorescence analysis and 15% dPAGE (Figure 5). The fluorescence intensity signal of CEM-Vh was the highest. In the presence of other pathogenic bacteria as target, cleavage of DNAzyme was not observed, and the fluorescence remained the same. These observations indicated the good specificity of DVh_3+1_.

### 3.4. Sensitivity of DVh_3+1_

**Figure 6:**
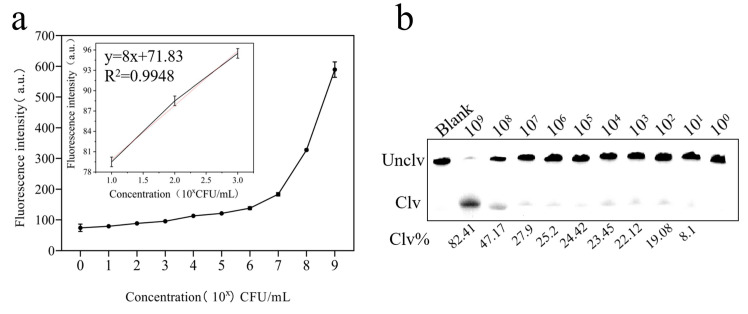
Sensitivity of DVh_3+1_: (**a**) fluorescence intensity signals of different concentrations of *V. harveyi* (Blank: normal saline) and the calibration curves constructed using the fluorescence signals corresponding to 4.7 × 10^1^, 4.7 × 10^2^, and 4.7 × 10^3^ CFU/mL of *V. harveyi*; and (**b**) gel cleavage assay at different concentrations of *V. harveyi* (Blank: reaction system without CEM-Vh).

The number of colonies in the initial culture medium of *V. harveyi* was 4.7 × 10^9^ CFU/mL. The culture method used has been described in 2.1. As shown in Figure 6a, the cleavage activity of DVh_3+1_ (indicated by the fluorescence intensity) gradually increased with an increase in the concentration of *V. harveyi* (10^0^–10^9^). The corresponding analytical calibration curve (y = 8x + 71.833, R^2^ = 0.9944) was plotted based on the fluorescence signals (Figure 6a). The limit of detection (LOD) was 27 CFU/mL, which was calculated as follows:

LOD = (K × Sb/m)

where K is a coefficient determined at a certain confidence level (taken as 3), Sb is the standard deviation of blank (1.527), and m is the slope of the analytical calibration curve in the concentration range of 10^1^ to 10^3^. The background concentration of *V. harveyi* was 4.7 × 10^1^ CFU/mL.

Cleavage bands and the percentage of DVh_3+1_ band both showed that the cleavage rate of DVh_3+1_ decreased with the decrease in the concentration of *V. harveyi* (Figure 6b). When the concentration of *V. harveyi* was 4.7 × 10^1^ CFU/mL, cleavage of band was still observed. The above results indicated that the sensitivity of DVh_3+1_ was good and that it could be used for preparation of the sensor.

### 3.5. Identification of the Target of DVh_3+1_

**Figure 7:**
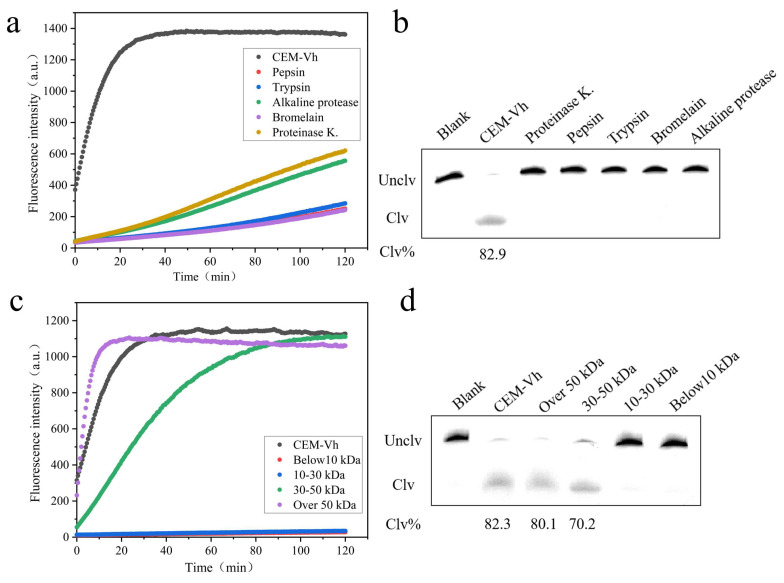
Identification of the target of DVh_3+1_: (**a**) fluorescence intensity of untreated and protease-treated CEM-Vh cleaved by DVh_3+1_; (**b**) 15% dPAGE analysis of the cleavage activity of DVh_3+1_ against CEM-Vh treated with various proteases; (**c**) fluorescence intensity of the reactions between DVh_3+1_ and CEM-Vh with different molecular weights; (**d**) 15% dPAGE analysis of the cleavage activity of DVh_3+1_ against CEM-Vh with different molecular weights. (Blank: reaction system without CEM-Vh).

After 1 h treatment with proteinase K, pepsin, trypsin, bromelain, and alkaline protease, the proteins in CEM-Vh were degraded to different extents. As shown in Figure 7a, fluorescence intensities of the CEM-Vh treated with the five proteases were significantly different than the untreated CEM-Vh, which confirmed that the target of DVh_3+1_ was protein. After 1 h, fluorescence intensities of the protease-treated CEM-Vh samples started to increase, which may be due to the cleavage of remaining non-degraded proteins in CEM-Vh by DVh_3+1_. Figure 7c shows the fluorescence of the DVh_3+1_ reaction with CEM-Vh of different molecular weights. CEM-Vh filtrates with 30–50 kDa and over 50 kDa molecular weights could be cleaved by DVh_3+1_, producing fluorescence. On the other hand, filtrates with less than 30 kDa could not produce a fluorescence intensity signal. The results of 15% dPAGE, shown in Figure 7d, further verified this finding. These results suggested that the protein recognized by DVh_3+1_ was not a single protein and DVh_3+1_ could cleave the proteins with molecular weights of 30–50 kDa and over 50 kDa in CEM-Vh.

### 3.6. Effects of RNases

**Figure 8:**
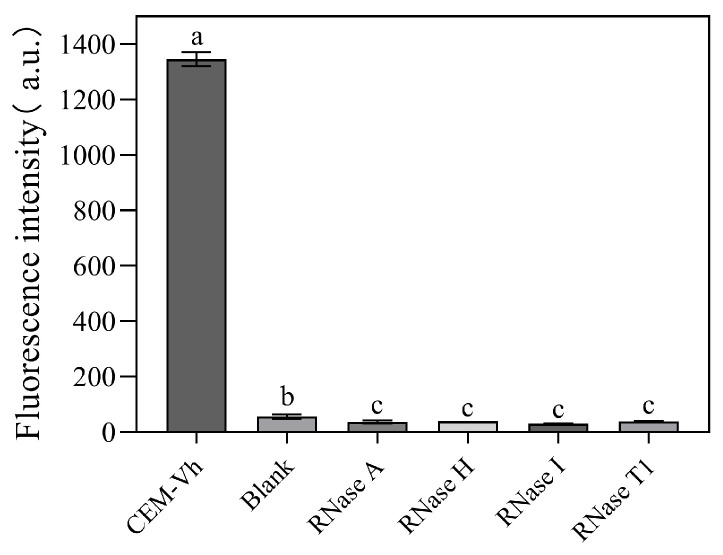
Effect of four different RNases on the cleavage activity of DVh_3+1_ (Blank: reaction system without CEM-Vh; Different letters indicate statistically significant differences (*p* < 0.05), while identical letters indicate insignificant differences (*p* > 0.05)).

Since it was not possible to purchase an RNase for *V. harveyi*, commercial RNases obtained from *E. coli* were purchased and used in this experiment. As shown in Figure 8, DVh_3+1_ exhibited little cleavage activity in presence of RNases.

### 3.7. Design of Dual DNAzyme DVh_3+1_ Sensor

**Figure 9:**
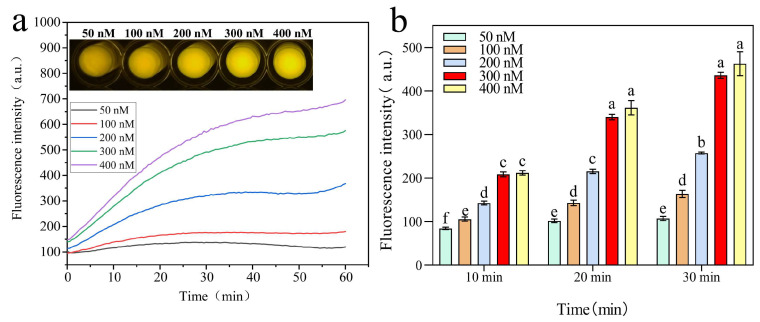
(**a**) Optimization of the concentration of DVh_3+1_-S in the dual DNAzyme sensor, with corresponding pictures of fluorescence signal shown at the top. (**b**) Analysis of significant differences between the fluorescence values of different concentrations of DVh_3+1_-S at different reaction times. Different letters indicate statistically significant differences (*p* < 0.05), while the same letters indicate insignificant differences (*p* > 0.05). The bar represents the mean ± SD.

The optimization results of the dual DNAzyme sensor are shown in Figure 9a. The fluorescence signal was concentration-dependent, with higher concentrations yielding stronger signals. Also, the fluorescence signal increased over time. When the concentration of DVh_3+1_-S was 300 nM, obvious changes in fluorescence were observed in blue-light photographs and after the addition of target CEM-Vh to the sensor. Therefore, 300 nM was selected as the optimal concentration of DVh_3+1_-S. Moreover, at same reaction time, there was no significant difference in the fluorescence of DVh_3+1_-S at 300 nM and 400 nM concentrations. Considering cost reduction, 300 nM was selected as the optimal concentration. Figure 9b shows that the fluorescence intensity of 300 nM DVh_3+1_-S was highest at reaction time of 30 min. Therefore, 30 min was considered to be the optimal reaction time for the DVh_3+1_ sensor.

### 3.8. Detection of V. harveyi Infection in Four Actual Samples by Dual DNAzyme DVh_3+1_ Sensors

**Figure 10:**
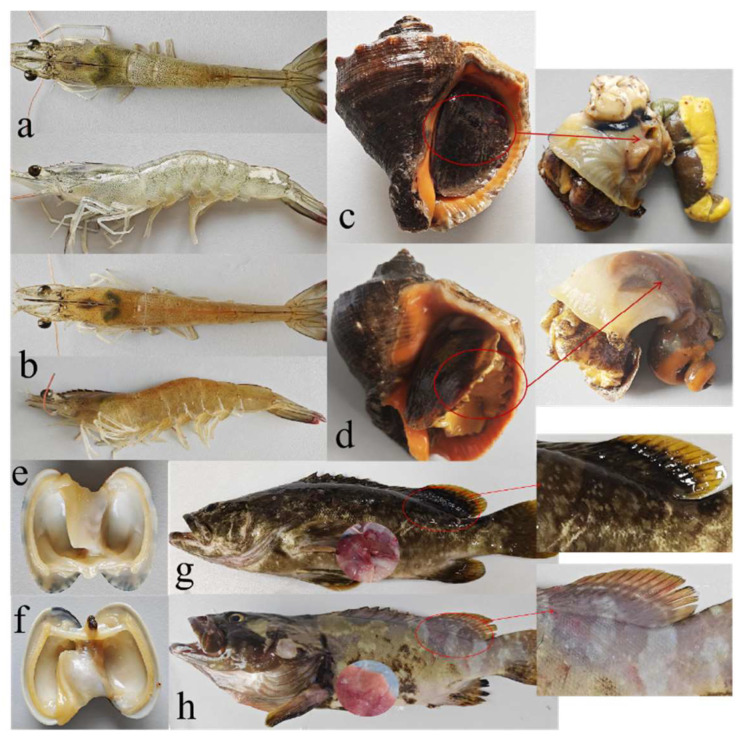
Comparisons of the aquatic products infected by *V. harveyi*: (**a**) *P. vannamei* before infection, (**b**) *P. vannamei* after infection 24 h, (**c**) whelk before infection, (**d**) Whelk after infection 60 h, (**e**) *C. formosana* before infection, (**f**) *C. formosana* after infection 48 h, (**g**) *Epinephelus* before infection and (**h**) *Epinephelus* after infection 50 h.

The aquatic organisms in the experimental groups began to die after 24 h. After 60 h, all organisms in the four experimental groups were dead. Figure 10 shows the body surfaces of the organisms in the control and experimental groups. The body surface of the *Epinephelus* infected with *V. harveyi* showed obvious symptoms, such as scale shedding, pus, and eye lesions. Infected *Penaeus vannamei* also showed the symptoms of red-body disease. The lesions were consistent with the symptoms of *V. harveyi* infection. However, no obvious lesions were observed on the bodies of whelk and *Cyrenodonax formosana*. The DNA sequences obtained from the four organisms were compared with the sequence of *V. harveyi.* The results revealed 99.99% similarity between the sequences, which confirmed *V. harveyi* infection in the four organisms.

**Figure 11:**
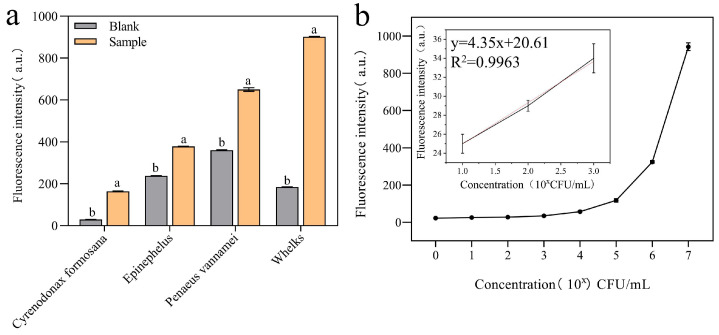
*V. harveyi* detection in the actual samples by DVh_3+1_ sensor: (**a**) fluorescence intensities of blank and four actual samples (*C. formosana*, *Epinephelus*, *P. vannamei*, and whelk; Blank: uninfected animal samples); (**b**) fluorescence intensities of diluted whelk samples, with calibration curves plotted using the fluorescence values of *V. harveyi* at concentrations of 1.02 × 10^1^, 1.02 × 10^2^, and 1.02 × 10^3^ CFU/mL (Different letters indicate statistically significant differences (*p* < 0.05)).

As shown in Figure 11, DVh_3+1_ showed obvious fluorescence intensity signals in the samples of all four organisms infected with *V. harveyi*, with whelk showing the highest fluorescence intensity. The whelk group had a lower blank fluorescence background. Therefore, whelk infected with *V. harveyi* were selected for subsequent detection experiments as described in 2.8. The LOD of sensor for whelk was calculated to be 11 CFU/mL (Figure 11b). This simple sensor can be used for on-site, rapid detection of bacteria.

## 4. Discussion

*V. harveyi* is an important pathogenic bacterium in aquaculture. Therefore, developing a rapid detection method is important for controlling the diseases caused by *V. harveyi* [3]. In addition to the traditional detection methods, which are complicated and time-consuming, some new methods have been reported for detection of *V. harveyi*. Catia et al. used real-time qPCR to detect *V. harveyi* and compared the influence of DNA extraction efficiency on its detection limit, which reached 48 CFU/mL [39]. Furthermore, Sithigorngul et al. used immunochromatographic coupled colloidal gold for rapid detection of *V. harveyi* [40]. This method was simple and easy to operate, but its sensitivity was not good (10^6^ CFU/mL). In addition, multiplex PCR assay [41], LAMP assay, and other methods have also been applied for rapid detection of *V. harveyi* [42]. However, these methods are expensive, rely on special instruments and equipment, and require professional laboratory environment and testing personnel, which limits the applicability of these methods for on-site detection.

In 2020, Setlem introduced new dual-fit DNAzyme for detection of AFB_1_ in complex samples with high stability and specificity [37]. Aptamer and DNAzyme are functional nucleic acids which are widely used for detection due to their high specificity, good sensitivity, low cost, and miniaturization [14]. Therefore, a dual DNAzyme DVh_3+1_ florescent sensor was constructed in this study for rapid detection of *V. harveyi* in aquatic products. DNAzymes were screened by the magnetic-bead method. Furthermore, positive and negative screening was conducted for CEM-Vh and other bacterial CEMs to improve the specificity of the selected DNAzyme. DVh_3+1_ was selected based on sequencing results and comparisons of cleavage activity, specificity, and sensitivity.

Sun et al. found that CEM was the main pathogenic component of *V. harveyi*, and contained proteins, nucleic acids, lipids, and polysaccharides [43]. Many reports have suggested that protein is the main target of DNAzyme in CEM [23,24,27]. Therefore, the proteins in CEM-Vh were degraded using five proteases. The results revealed that these proteases failed to cleave DNAzyme, confirming that the proteins were the target of DVh_3+1_. The filter test showed that DNAzyme could simultaneously capture proteins of 30 kDa, 50 kDa, and over-50 kDa weights in CEM-Vh, thus showing improved detection efficiency.

## 5. Conclusions

In summary, two DNAzymes, DVh_3_ and DVh_1_, with good specificity and sensitivity to *V. harveyi*, were successfully screened in vitro. These DNAzymes were used to develop and optimize dual DNAzyme biosensors for the rapid detection of *V. harveyi*. The sensor’s detection limit was as low as 11 CFU/mL and the detection results were observed within 30 min. Therefore, this dual DNAzyme biosensor can be used for the on-site detection of *V. harveyi*, which would be helpful in timely prevention and control of *V. harveyi* infection in aquaculture, thereby reducing the risk to human health and promoting the healthy and stable development of the aquaculture industry.

## Figures and Tables

**Table 1:** DNA sequences used in this study.

Name	Oligonucleotide Sequences (5′-3′)
Lib	Phosp-GACATTACGGGGGCGTAATG-N40-GCATCTGTAGCGTAGTGTCG
FP	Biotin-CTACAGATGCTrAGACATTACGGGGGC
RP	CGACACTACGCTACAGATGC
DVh_1_	GCCCCCGTAATGTCATGTAGCCGCAGGCGCCATCATGTTGCACCCTAAGGGATTGCATCTGTAGCGTAGTGTCG-Q
DVh_3_	GCCCCCGTAATGTCAAGGTCCCGCAGGGGCTTTCACTTCGTCCCTGTATGTTTCGCATCTGTAGCGTAGTGTCG-Q
Substrate	FAM-CGACACTACGCTACAGATGCTrAGACATTACGGGGGC

Note: Library and primer sequences have been listed here. Abbreviations: Lib, library; FP, forward primer; RP, reverse primer; Phosp, phosphorylation; N40, 40 random nucleotides; rA, adenosine ribonucleotide; Q, quencher; FAM, fluorophore.

**Table 2:** Sequences of the six candidate DNAzymes.

Name	Number of Sequences	Percentage of Total Sequence	Randomized Sequence of Regions (N40, 5′-3′)
DVh_1_	23824	26.17%	ATGTAGCCGCAGGCGCCATCATGTTGCACCCTAAGGGATT
DVh_2_	20002	21.97%	ACTAATGTGCGAAGCTCGTTAGTTCTACGCACGCGTAATG
DVh_3_	9739	10.7%	AAGGTCCCGCAGGGGCTTTCACTTCGTCCCTGTATGTTTC
DVh_4_	6072	6.7%	GGGGCGCAACGCGCCTACCTTTCGACGTCCGGCGATGTTA
DVh_5_	2961	3.25%	AGGAAAGGAACTGCGCTCGGTCGACCTTAACGTAGTGGCC
DVh_6_	1507	1.66%	ACTAACGTGCGAAGCTCGTTAGTTCTACGCACGCGTAATG

## Data Availability

The interaction data used to support the study findings are included in this article. Moreover, all the data used to support the study findings are available from the corresponding author upon request.
